# Structural and physicochemical effects on the starch quality of the high-quality wheat genotype caused by delayed sowing

**DOI:** 10.3389/fnut.2024.1389745

**Published:** 2024-04-16

**Authors:** Xiaomei Huang, Xin Zhou, Xueqing Liu, Wen Zhong, Xinyu Wang, Zhengchun Ju, Yan Yin, Qingguo Xin, Ning Liu, Ximei Liu, Yuli Jin, Guie Wang, Jiangchun Wang, Pengtao Ma

**Affiliations:** ^1^Yantai Key Laboratory of Characteristic Agricultural Biological Resources Conservation and Germplasm Innovative Utilization, College of Life Sciences, Yantai University, Yantai, China; ^2^Institute of Grain and Oil Crops, Yantai Academy of Agricultural Sciences, Yantai, China; ^3^Shandong Seed Administration Station, Jinan, China; ^4^Shandong Agricultural Technology Extension Center, Jinan, China; ^5^Shandong Zhongnong Tiantai Seed Industry Co., Ltd., Linyi, China

**Keywords:** wheat, sowing time, starch properties, starch synthase, agronomic and yield performance

## Abstract

**Background:**

Bread wheat is one of the most important food crops associated with ensuring food security and human nutritional health. The starch quality is an important index of high-quality wheat. It is affected by a complex series of factors; among which, suitable sowing time is a key factor.

**Aim and methods:**

To analyze the integrative effects of sowing time on the starch quality of high-quality wheat, in the present study, we selected a high-quality bread wheat cultivar Jinan 17 and investigated the effect of different sowing times on the starch properties and the related genes by analyzing X-ray diffraction patterns, apparent amylose content, thermal properties, pasting properties, *in vitro* starch digestibility, and qRT-PCR. Meanwhile, we also investigated the agronomic and yield performance that may be associated with the starch properties.

**Results:**

Delayed sowing had little effect on starch crystalline morphology, but there was a tendency to reduce the formation of crystals within wheat starch granules: (1) delayed sowing for 15 days altered the thermal properties of starch, including onset, peak and termination temperatures, and enthalpy changes; (2) delayed sowing for 30 days changed the thermal characteristics of starch relatively insignificant; (3) significant differences in pasting characteristics occurred: peak viscosity and hold-through viscosity increased, while final viscosity, breakdown viscosity, and setback viscosity tended to increase and then decrease, suggesting that delayed sowing caused changes in the surface of the starch granules resulting in a decrease in digestibility. Analysis of related genes showed that several key enzymes in starch biosynthesis were significantly affected by delayed sowing, leading to a reduction in apparent straight-chain starch content. In addition to starch properties, thousand-kernel weight also increased under delayed sowing conditions compared with normal sowing.

**Conclusion:**

The impact of delayed sowing on starch quality is multifaceted and complex, from the fine structure, and functional properties of the starch to the regulation of key gene expression. Our study holds significant practical value for optimizing wheat planting management and maximizing the potential in both quality and yield.

## 1 Introduction

Bread wheat (*Triticum aestivum* L.) is one of the most important food crops that provide 35% of the total calories required by humans and hence was closely associated with ensuring food security and human nutritional health worldwide (1, 2). In wheat flour, starch accounts for approximately 70–80% and is the predominant carbohydrate and the most abundant renewable polysaccharide in cereals after cellulose in the wheat grain. Hence, it has a significant influence on both the functional properties and processing quality of the flour (3). However, starch composition and quality vary according to environmental and genetic factors. For instance, they are sensitive to heat and drought stresses, which have a significant impact on the seed filling and flowering periods, which can negatively affect the starch synthesis (4); different genotypes also have diversified starch composition and functionality, such as pasting, thermal, and digestive properties. In starch composition, morphology and structure are closely related to starch functionality, which, in turn, is influenced by the genetic basis and growth environment (1).

At the molecular level, starch biosynthesis is a delicate and complex process that requires meticulous coordination among multiple enzymes. As for the biosynthesis of wheat starch, the key enzymes mainly include granule-bound starch synthase (GBSS) and the glucosyltransferase enzyme, which is primarily responsible for the synthesis of straight-chain starch (5). The process of straight-chain starch synthesis itself is relatively simple, with linear chains being formed by the continuous stretching action of one enzyme, GBSS (5). However, this process must be closely coordinated with the activity of the starch branching enzyme (SBE) to form granules with the correct structure and composition (6). Furthermore, other enzymes, such as starch synthase (SS), SBE, and starch debranching enzyme (DBE), are responsible for the process of starch chain lengthening, branching, and debranching, respectively (6). Isoamylase (ISA) removes the improper branches of amylopectin and forms a highly ordered structure, which helps amylopectin to form a stable cluster structure. There is a significant relationship between the expression of *ISA1* and amylopectin (7–9). Pullulanase (PUL) typically cleaves the α-1,6-bonds of the polyglucan in branched-chain starches and, to a lesser extent, branched-chain starches and is virtually inactive on glycogen (4, 6, 10, 11). In these processes, enzyme activity and expression are often influenced by genetic and environmental factors, especially under high temperature and drought conditions, and the changes in enzyme activity and the gene expression level can significantly affect starch synthesis and accumulation. For example, if the wheat is prematurely or untimely exposed to high temperatures during the flowering and filling periods, the starch synthesis and accumulation process will be severely disrupted (12). Previous studies have shown that the expression of 22 genes related to starch synthesis was downregulated and only one *ISA2* gene was upregulated under high-temperature treatment (13). In addition, drought stress upregulated the expression of 17 genes related to starch synthesis, while downregulated the expression of six others (including the granule-bound starch synthase *GBSSI*, *AGPS2*, *BEIIb*, *PHOL*, *ISA1*, and *AGPL2*) (14, 15). These changes affect starch synthesis and accumulation, resulting in lower amylose content.

In addition, untimely high temperature also causes significant changes in starch functionality mainly in tropical and temperate cereals. Among them, the content of amylose is particularly sensitive to changes in environmental conditions, which mainly affects the physicochemical properties of wheat starch (16). Starch with higher amylose content usually has lower peak viscosity (PV) and higher final viscosity (FV), setback viscosity (SBV), and pasting temperature (PT). Amylopectin is the main component of the increase in swelling power and viscosity of starch during gelatinization. For example, in wheat starch, the formation of viscosity is impelled when amylose and lipids form the helix complex. Therefore, amylose is negatively correlated with PV (17, 18). The gelatinization temperature is related to the stability and integrity of the crystalline structure. The amylose in the amorphous region inhibits the water absorption of starch granules, thereby increasing the gelatinization temperature (18, 19). In addition, starch consists of five structural levels, namely, molecular, crystalline, growth ring, lamellar, and granular structures (20); deeper dissection is necessary to clarify the regulatory mechanism caused by the environmental changes.

In recent years, global climate change has intensified, which has a serious impact on grain production (21). Due to global warming, the growth process of winter wheat is accelerated, which increases the possibility of freezing injury before winter and spring in the production process of wheat, and has a significant impact on yield and quality. Challenges are being encountered with the traditional planting time. Appropriately delaying the sowing time can cope with the adaptive strategy of unstable climate conditions. It is not clear whether delicate effects on the starch will be induced under different sowing times of the high-quality wheat cultivars. Therefore, in this study, we selected a high-quality wheat cultivar, Jinan 17, to comprehensively analyze the effects of delayed sowing conditions on the starch properties, including starch crystallinity, thermal properties, pasting characteristics, *in vitro* digestibility, and changes in expression patterns of the SS genes behind these phenotypic changes, as well as the agronomic and yield traits that may be associated with the starch properties. This study offers novel insights into the molecular mechanisms underlying the impact of delayed sowing on wheat starch quality, thereby informing future research endeavors and production practices.

## 2 Materials and methods

### 2.1 Plant material and sowing design

The wheat cultivar Jinan 17, developed by the Crop Research Institute, Shandong Academy of Agricultural Sciences, Jinan, China, is a high-quality wheat for making bread. Three sowing times, namely, normal sowing (NS) (24 October 2021, 8.9°C), delayed sowing for 15 days (DS1) (9 November 2021, 6.5°C), and delayed sowing for 30 days (DS2) (24 November 2021, 3.1°C), were set. The wheat cultivar Jinan 17 was planted in the National Crop Variety Regional Test Station in Yantai, China (37°65′59″N, 120°47′01″E). Each experimental design was planted on a plot with nine rows (1.5 m length and 0.25 m between rows), and three replications were carried out. Field management, such as watering, weeding, and other agronomic measures, for different plots were consistent throughout the whole growth period. When the plants were harvested at the mature period, the plants were randomly selected from each row and the samples were mixed to extract their starch. Three replications were also carried out for starch properties and agronomic and yield performance analysis.

### 2.2 Starch extraction

Starch was extracted from the harvested wheat grain referred to the reported procedure with minor modifications (22). Briefly, wheat kernels (300 g) were washed and soaked in 0.3% NaOH solution (1:3 w/v) for 24 h. After draining the water through a sieve, the kernels were poured into a homogenizer for crushing. After fully grinding, the powder was passed through a 70-mesh sieve. The filtrate was then centrifuged to remove the upper protein layer and retain the white starch layer at the bottom, and then, the sediment was mixed well by adding the appropriate amount of water to the centrifuge bottle and re-centrifuged. The procedure was repeated to remove the protein layer and then neutralized with hydrochloric acid to a neutral pondus. The filtrate was then diafiltrated, and the filter cake was washed three times with anhydrous ethanol before being centrifuged. The collected filter cake was then placed in an oven at 40°C for 48 h at a constant temperature. The dried filter cake was ground in a mortar and pestle, and the ground powder was sieved through a 100-mesh sieve. In the end, the powder was collected and stored in a sealed container for subsequent quality analysis.

### 2.3 X-ray diffraction

The X-ray pattern of the starch was analyzed using an X-ray powder diffractometer (Siemens D500, Siemens, Germany). The measurement was conducted with an accelerating voltage of 30 kV and a current of 30 mA. The scanning speed was 10°/min. The diffractogram was recorded within the 2*θ* range of 5–40°, with a sample width of 0.02° being employed. Relative crystallinity (RC) was quantitatively calculated following the MDI-Jade software (v6.0) (23).

### 2.4 Determination of the apparent amylose content

The apparent amylose content (AAC) in the starch was measured by the iodine reagent method (24). The absorbance of the solution was measured at 620 nm using a spectrophotometer and compared with a blank solution. AAC was calculated using a standard curve drawn from five samples with known AAC.

### 2.5 The thermal properties of the starch

The thermal properties of the starch were analyzed using a differential scanning calorimeter (DSC, Photo-DSC 204 F1 Phoenix, NETZSCH, Germany). Duplicate samples of 2 mg of wheat starch were weighed and mixed with 6 mL of distilled water in an aluminum pan. The pan was then hermetically sealed and equilibrated at −4°C for 24 h before thermal analysis. The temperature of the pan was increased at a rate of 10°C/min from 30°C to 100°C. A sealed container pan was used as a standard for comparison. The onset temperature (*T*_o_), peak temperature (*T*_p_), conclusion temperature (*T*_c_), and gelatinization enthalpy (Δ*H*) were then calculated (25).

### 2.6 Pasting properties

The pasting properties of the flour were measured using a Rapid Visco-Analyzer (RVA-4, Newport Scientific, and Warriewood, Australia) with standard profile I. To conduct the analysis, 3 g of the starch (14% moisture basis) was directly weighed into an aluminum RVA sample canister, followed by the addition of 25 mL of distilled water. The samples underwent a programmed heating and cooling cycle, commencing with a 10 s hold at 50°C and a speed of 960 rpm. The speed was then reduced to 160 rpm and maintained at 50°C for 1 min, followed by heating to 95°C for 3.7 min, and a subsequent hold at 95°C for 2.5 min. The samples were then cooled to 50°C over 3.8 min and held at that temperature for 2 min. The parameters recorded during the analysis included PV, hold-through viscosity (TV), breakdown viscosity (BDV), FV, SBV, and PT which referred to the reported method (26, 27).

### 2.7 *In vitro* starch digestibility

Rapidly digestible starch (RDS), slowly digestible starch (SDS), and resistant starch (RS) contents of the starch samples were determined using the reported methods with slight modifications (28, 29).

### 2.8 RNA isolation, cDNA synthesis, and qRT-PCR

Grain samples were collected from the central parts of the spikes at 5, 10, and 15 days after anthesis. Immediately after sampling, the kernels were frozen in liquid nitrogen for 15 min and then stored at −80°C for RNA extraction. Total RNA was extracted from the endosperm of the grain using a kit (Vazyme, R223, China) according to the manufacturer’s instructions. The RNA integrity was evaluated using the Agilent 2100 Bioanalyzer (Agilent Technologies, Santa Clara, CA, United States). Samples with an RNA Integrity Number (RIN) ≥7 were regarded as meeting the standard of inverse transcription. cDNA was synthesized using a transcription kit (Vazyme, R223, China), and the quality of the cDNA checked was again assessed using the Agilent 2100 Bioanalyzer (Agilent Technologies, Santa Clara, CA, United States), and the acceptable cDNA was used for subsequent qRT-PCR. The reaction solution was prepared using the ChamQ Universal SYBR qPCR Master Mix Kit (Vazyme, Q711, China). qRT-PCR was carried out on the Bio-Rad CFX Connect real-time PCR system (BIO-RAD, United States). For each sample, the gene *TaActin* was used as the internal control for normalization. Three technical replications were analyzed.

### 2.9 Evaluation of agronomic traits

The wheat cultivar Jinan 17 was planted in 2021–2022 at the Yantai National Regional Experiment Station for Crop Varieties (37°65′59″N, 120°47′01″E), Yantai, China, with three replications for each sowing period. Each sowing period was planted in six rows (1.5 m in length and 0.25 m between rows) with 20 seeds per row. When the plants matured in June, 10 plants in the middle of the rows were randomly selected to evaluate their plant height (PH), spike length (SL), number of spikes per plant (SNPP), number of spikelets per spike (SNS), number of kernels per spike (KNS), and thousand-kernel weight (TKW). PH, SL, SNPP, SNS, and KNS were assessed using the mean values determined from selected plants. TKW was assessed by weighing a sample of 500 kernels of seeds with three replications.

### 2.10 Statistical analysis

All data were collected in three replicates, and the results were presented as the mean ± standard deviation. The statistical analyses were conducted using SPSS software (version 26.0, SPSS, Chicago, IL, United States) with a statistical significance level of 0.05.

## 3 Results

### 3.1 X-ray pattern of the starch in different sowing dates

**Figure 1:**
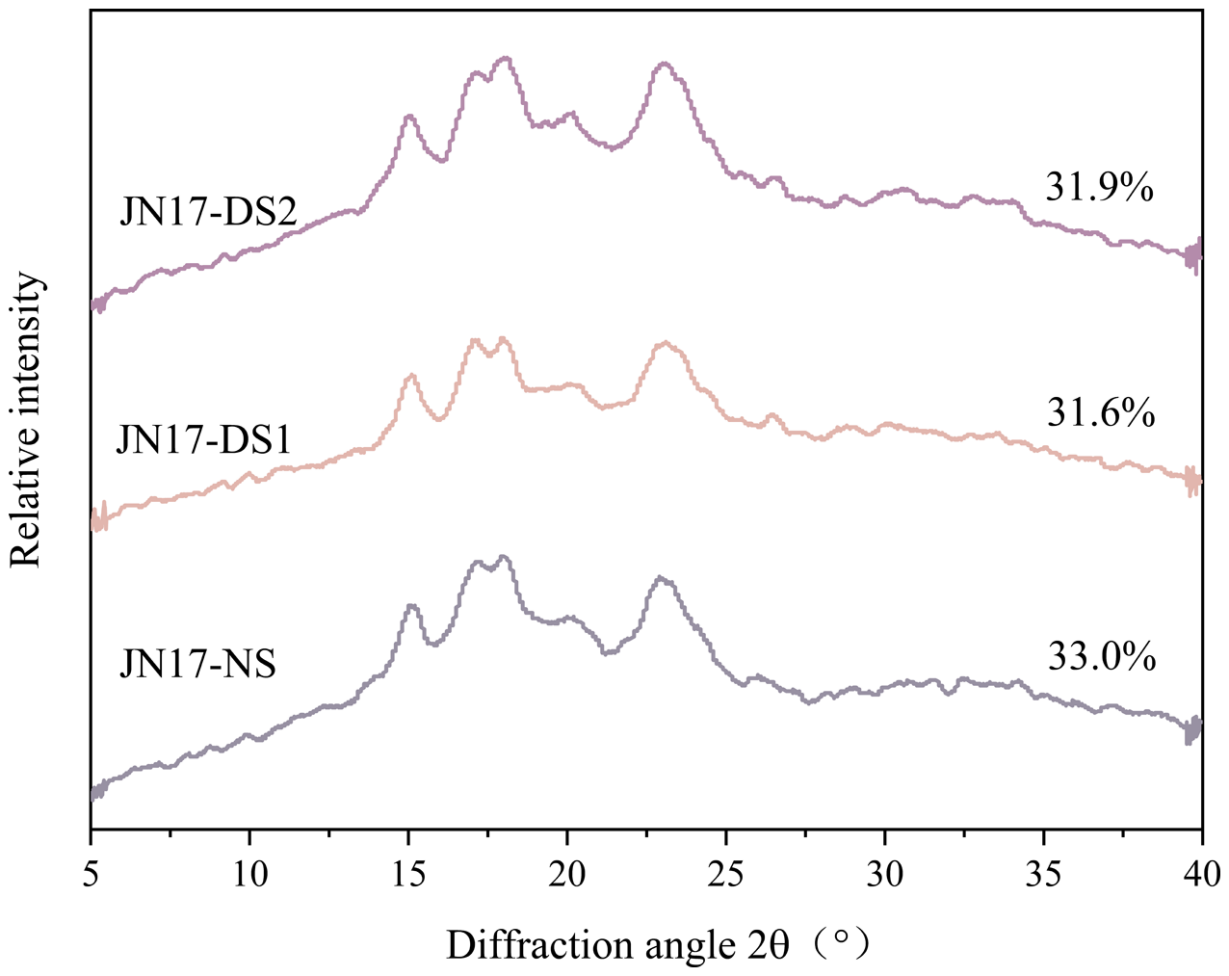
X-ray diffraction patterns of the wheat cultivar Jinan 17 (JN17) starch samples under normal sowing (NS), delayed sowing for 15 days (DS1), and delayed sowing for 30 days (DS2).

After X-ray diffraction, the long-range ordered structure of the starch double helix was determined, which can reflect the three-dimensional ordering of starch crystals. The result demonstrated that the starches under DS1 and DS2 had diffraction peaks near 15° and 23°, respectively, strong diffraction bimodal peaks near 17° and 18°, and weak diffraction peaks both near 20°, which were the typical characteristic of the A-type starch (Figure 1 and Table 1). Compared with NS, the RC of the starch under DS1 was lower and the RC of starch under DS2 was lower than that under DS1. The crystals of the starch had a relatively high intensity of the spikes approximately 2θ = 15° and 2θ = 17° and the relatively low intensity of the spikes approximately 2θ = 20° and 2θ = 23°, suggesting that delayed sowing had no significant effect on the crystal type of starch, which would lead to the change of RC of starch. The starch RC decreased with the delay of the sowing date, and the decreasing trend of DS1 and DS2 was similar. The results showed that delayed sowing did not change the crystal type of wheat starch, but affected the relative crystallinity.

**Table 1:** The determination and analysis of the apparent amylose content (AAC) and relative crystallinity (RC), rapidly digestible starch (RDS), slowly digestible starch (SDS), and resistant starch (RS) contents under the condition of normal sowing (NS), delayed sowing for 15 days (DS1), and delayed sowing for 30 days (DS2) in the wheat cultivar Jinan 17.

Treatment condition	AAC (%)	RC (%)	RDS (%)	SDS (%)	RS (%)
NS	35.6 ± 0.0^a^	33.0 ± 1.3^a^	60.6 ± 0.7^a^	9.8 ± 0.1^a^	29.5 ± 0.9^c^
DS1	27.5 ± 0.0^c^	31.6 ± 0.2^a^	59.1 ± 0.5^a^	7.5 ± 0.0^b^	33.3 ± 0.5^b^
DS2	31.0 ± 0.0^b^	31.9 ± 0.0^a^	59.3 ± 0.0^a^	3.8 ± 0.5^c^	36.8 ± 0.5^a^

Means with the different superscripts in a column differ significantly (*p* ≤ 0.05). The displayed results are the means of three independent assays. Data represented as mean value ± SD based on the *chi*-squared test.

### 3.2 AAC analysis of the starch in different sowing dates

Amylose plays an important role in the properties of the starch. The content of AAC is closely related to the processing quality of starch. Compared with NS, the AAC content of DS1 (27.5%) and DS2 (31.0%) showed a downward trend, and the AAC content of DS2 was higher than that of DS1 (Table 1). This shows that the appropriate delayed sowing date will cause a decrease in AAC content in wheat, but this decreasing trend is limited. With the continuous delay of the sowing date, this trend will weaken or even disappear.

### 3.3 Thermal properties analysis of wheat starch in different sowing dates

**Figure 2:**
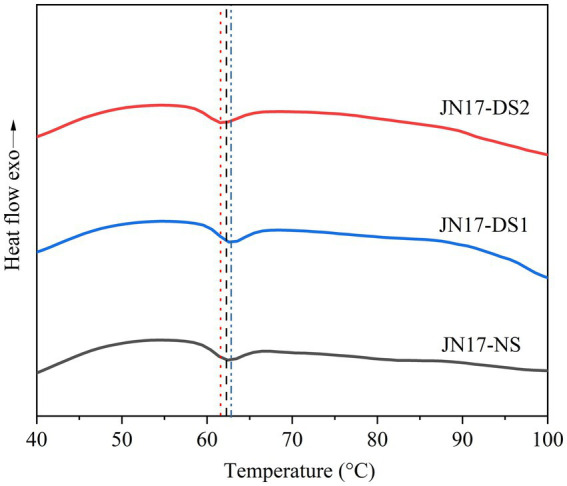
The pasting properties of the wheat cultivar Jinan 17 (JN17) starch samples under normal sowing (NS), delayed sowing for 15 days (DS1), and delayed sowing for 30 days (DS2).

Compared with NS, the T_o_ of the DS1 starch (58.4°C) was significantly lower than that of the NS (60.0°C), while the T_o_ of the DS2 starch (60.7°C) was slightly higher than that of DS1 (Table 2 and Figure 2). The T_p_ and T_c_ of the NS starch were slightly higher than those of the DS1 starch at 62.7°C and 65.3°C (Table 2 and Figure 2), respectively. DS2 starch had the highest T_o_ (60.7°C), T_p_ (63.3°C), and T_c_ (65.7°C). Interestingly, the ΔH of the DS1 starch was higher than that of the NS and DS2 starches, but the difference did not reach significance (Table 2 and Figure 2). This suggests that delayed sowing decreases the T_o_, T_p_, and T_c_ temperatures of the wheat starch and increases the ΔH, but the decreasing and increasing trends were not infinite, and it weakens or even disappears with the continued delay in sowing.

**Table 2:** Thermal properties of the starch of the wheat cultivar Jinan 17 under normal sowing (NS), delayed sowing for 15 days (DS1), and delayed sowing for 30 days (DS2).

Treatment condition	*T*_o_ (°C)	*T*_p_ (°C)	*T*_c_ (°C)	Δ*H* (J/g)
NS	60.0 ± 0.8^a^	62.7 ± 0.6^a^	65.3 ± 0.5^a^	1.2 ± 0.0^a^
DS1	58.4 ± 0.5^b^	61.6 ± 0.0^b^	63.7 ± 1.7^a^	1.5 ± 0.1^a^
DS2	60.7 ± 0.6^a^	63.3 ± 0.3^a^	65.7 ± 0.2^a^	1.2 ± 0.2^a^

Means with the same superscript in a column differ significantly (*p* ≤ 0.05). Displayed results are the means of two independent assays. Data represented as mean value ± SD based on the *chi*-squared test. *T*_o_, onset temperature; *T*_p_, peak temperature; *T*_c_, conclusion temperature; and Δ*H*, gelatinization enthalpy.

### 3.4 Analysis of pasting properties of starch in different sowing dates

Pasting properties are the core of the starch functionality. The analysis of RVA allowed the assessment of the viscosity parameters of the starch samples under delayed sowing (Table 3). The result indicated that PV and TV showed an increasing trend under delayed sowing. However, FV, BDV, and SBV showed an increasing and then decreasing trend under DS1 and DS2, with DS1 having the largest values of FV (3531.7 cP), BDV (500.2 cP), and SBV (1044.0 cP). This indicates that delayed sowing has an effect on the PV, TV, FV, BDV, and SBV of the wheat starch. Delayed sowing elevated the pasting characteristics of the wheat, but FV, BDV, and SBV of the wheat starch started to decrease with continued delay in sowing. These indicated that appropriate late sowing can improve the pasting characteristics of the starch.

**Table 3:** The pasting properties of the starch of the wheat cultivar Jinan 17 under normal sowing (NS), delayed sowing for 15 days (DS1), and delayed sowing for 30 days (DS2).

Treatment condition	PV (cP)	TV (cP)	FV (cP)	BDV (cP)	SBV (cP)
NS	2666.6 ± 40.5^b^	2229.0 ± 66.4^a^	3053.0 ± 33^b^	437.6 ± 51.3^a^	824.0 ± 64.5^a^
DS1	2988.0 ± 36.8^ab^	2595.7 ± 75.6^a^	3531.7 ± 40.5^ab^	500.2 ± 79.4^a^	1044.0 ± 28.6^a^
DS2	3056.3 ± 37.0^a^	2605.3 ± 125.5^a^	3453.0 ± 70.2^a^	451.0 ± 16.6^a^	847.6 ± 21.3^a^

Means with the same superscript in a column differ significantly (*p* ≤ 0.05). Displayed results are the means of two independent assays. Data represented as mean value ± SD based on the *chi*-squared test. PV, peak viscosity; TV, hold-through viscosity; BDV, breakdown viscosity; FV, final viscosity; SBV, setback viscosity; PT, pasting temperature.

### 3.5 *In vitro* digestion analysis of starch in different sowing dates

Compared with NS starch, the RDS and SDS of DS1 and DS2 starches decreased slightly, and the RS content increased to 33.3 and 36.8%, respectively (Table 1). The RDS and RS of starch of DS2 were slightly higher than those of DS1 (Table 1). These results indicated that delayed sowing can reduce the *in vitro* digestibility of the starch.

### 3.6 Expression patterns of SS genes in different sowing dates

**Figure 3:**
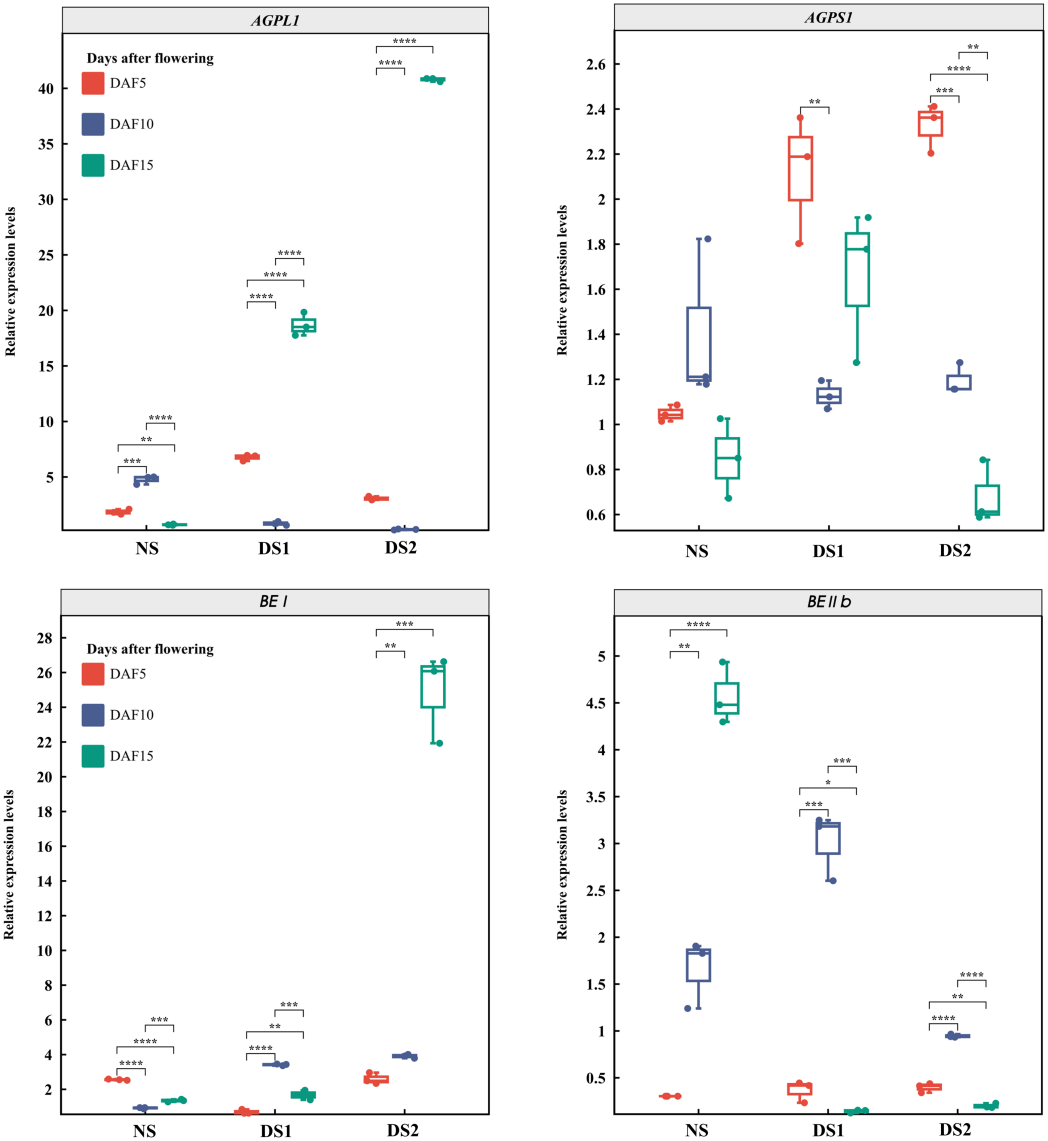
The expression patterns of the starch synthase (SS) genes *AGPL1*, *AGPS1*, *BEI*, and *BEIIb* in 5 (DAF5), 10 (DAF10), and 15 (DAF15) days after flowering.

**Figure 4:**
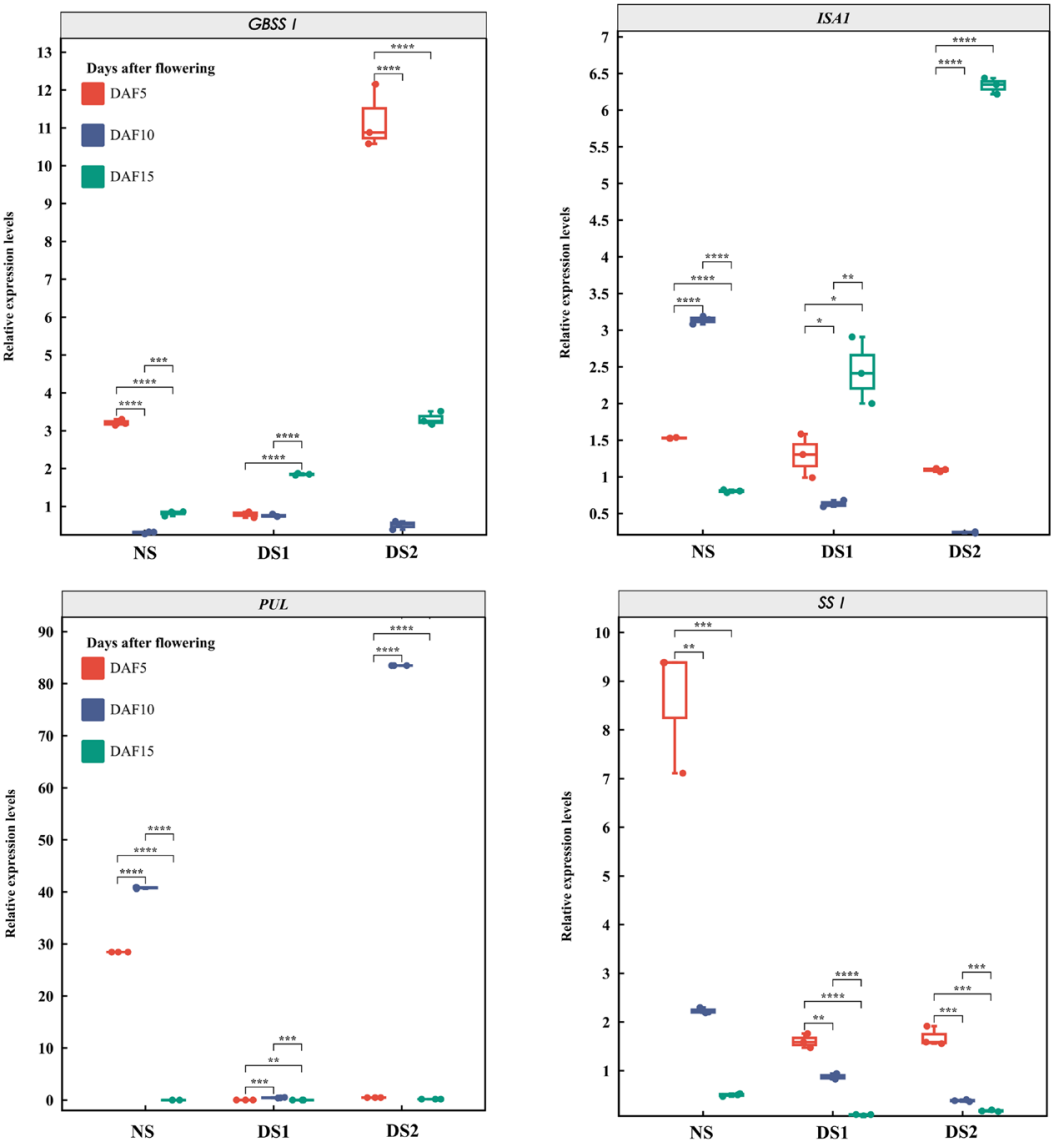
The expression patterns of the starch synthase (SS) genes *GBSSI*, *ISA1*, *PUL*, and *SSI* in 5 (DAF5), 10 (DAF10), and 15 (DAF15) days after flowering.

**Figure 5:**
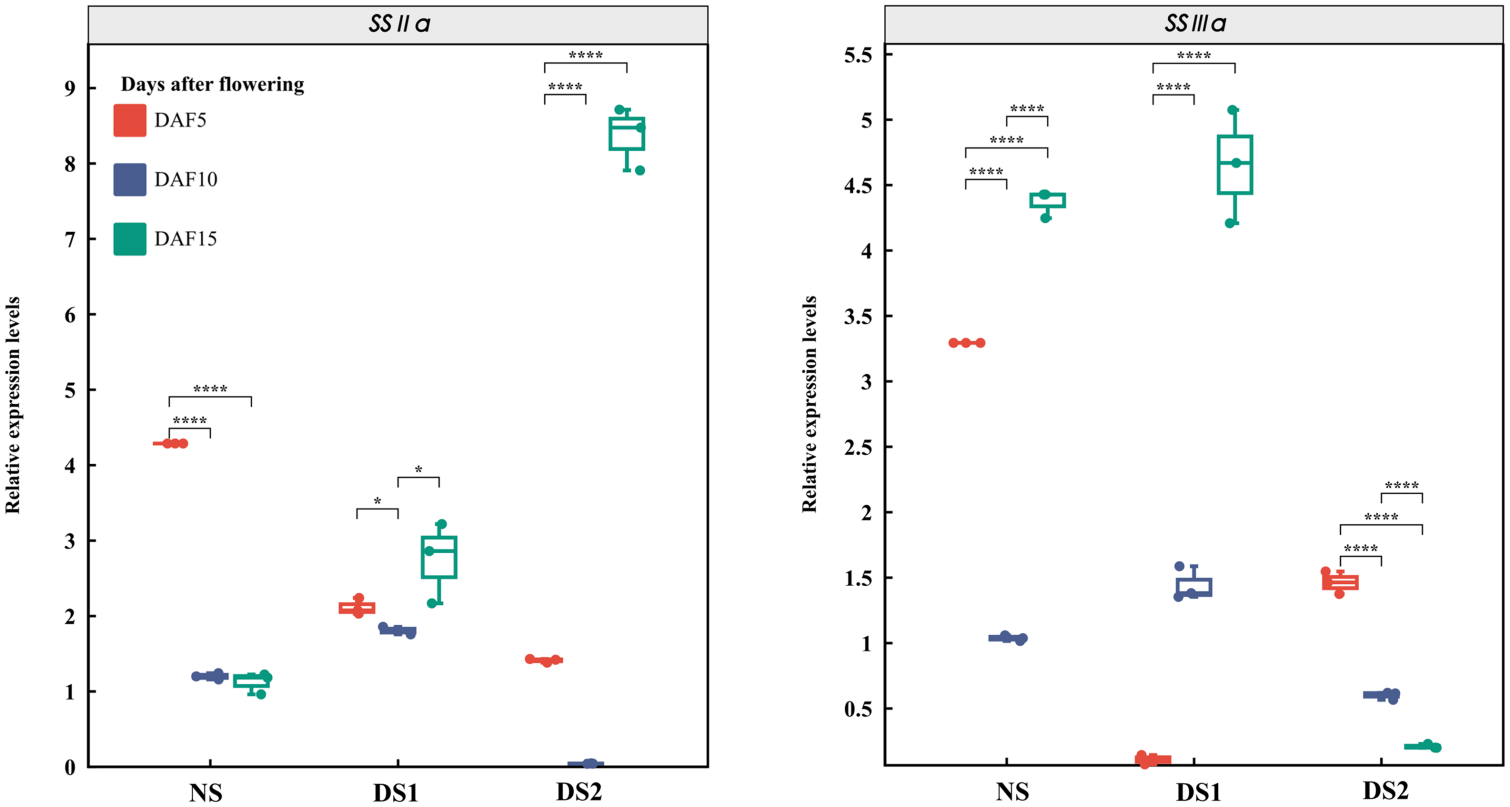
The expression patterns of the starch synthase (SS) genes *SSIIa* and *SSIIIa* in 5 (DAF5), 10 (DAF10), and 15 (DAF15) days after flowering.

In total, 10 SS genes and their expression patterns under different sowing times were selected (Figures 3–5). The results demonstrated that (1) the expression of *AGPL1* was upregulated at 5 and 15 days after anthesis and downregulated at 10 days after anthesis in DS1 and DS2 compared with those of NS; (2) the expression of *AGPS1* at 5 and 15 days after anthesis showed a trend of increasing and then decreasing with delayed sowing, while the expression at 10 days after anthesis showed the opposite trend; (3) the effect of DS1 on the expression of *BEI* was not significant, while the expression of the *BEI* gene in DS2 was significantly upregulated at 15 days after flowering; (4) in DS1 and DS2 treatments, the expression of *BEIIb* did not change much at 5 days after anthesis, increased first and then decreased at 10 days after anthesis, and decreased at 15 days after anthesis; (5) the expression of the *GBSSI* gene in DS1 and DS2 showed a trend of decreasing at first and then increasing at 5 days after flowering. There was no significant change in gene expression at 10 days after flowering, but it showed an increasing trend at 15 days after flowering; (6) the expression of *SSI* peaked at 5 days after flowering in all three sowing periods, and generally decreased in DS1 and DS2 treatments at sowing dates showing a trend of decreasing and then increasing gene expression at 5 days after flowering in DS1 and DS2, whereas the change in expression was not significant at 10 days after flowering, and then showed a gradual increase in expression at 15 days after flowering; (7) in DS1 and DS2, the expression of *ISA1* showed a gradually decreasing trend at 5 and 10 days after flowering, while the expression shows an increasing at first and then decreasing trend at 15 days after flowering; (8) compared with NS, the expression of *PUL* and *SSI* genes showed a downward trend under DS1 and DS2 treatments. The expression of *PUL* and *SSI* genes showed a gradual downward trend at 5 and 10 days after flowering. The expression of the *PUL* gene decreased first and then increased at 10 days after flowering, and the expression of the *SSI* gene decreased at 15 days after flowering; (9) under delayed sowing, the expression of the *SSIIIa* gene showed a decreasing and then increasing trend at 5 days after anthesis, and a trend of increasing and then decreasing at 10 and 15 days after anthesis. In conclusion, under delayed sowing, 10 key genes related to SS, including *AGPS1*, *AGPL1*, *BEI*, *BEIIb*, *GBSSI*, *ISA1*, *PUL*, *SSI*, *SSIIa*, and *SSIIIa*, were significantly induced to upregulate or downregulate expressions, which can be considered to respond to the delayed sowing.

### 3.7 Agronomic and yield performance in different sowing dates

**Figure 6:**
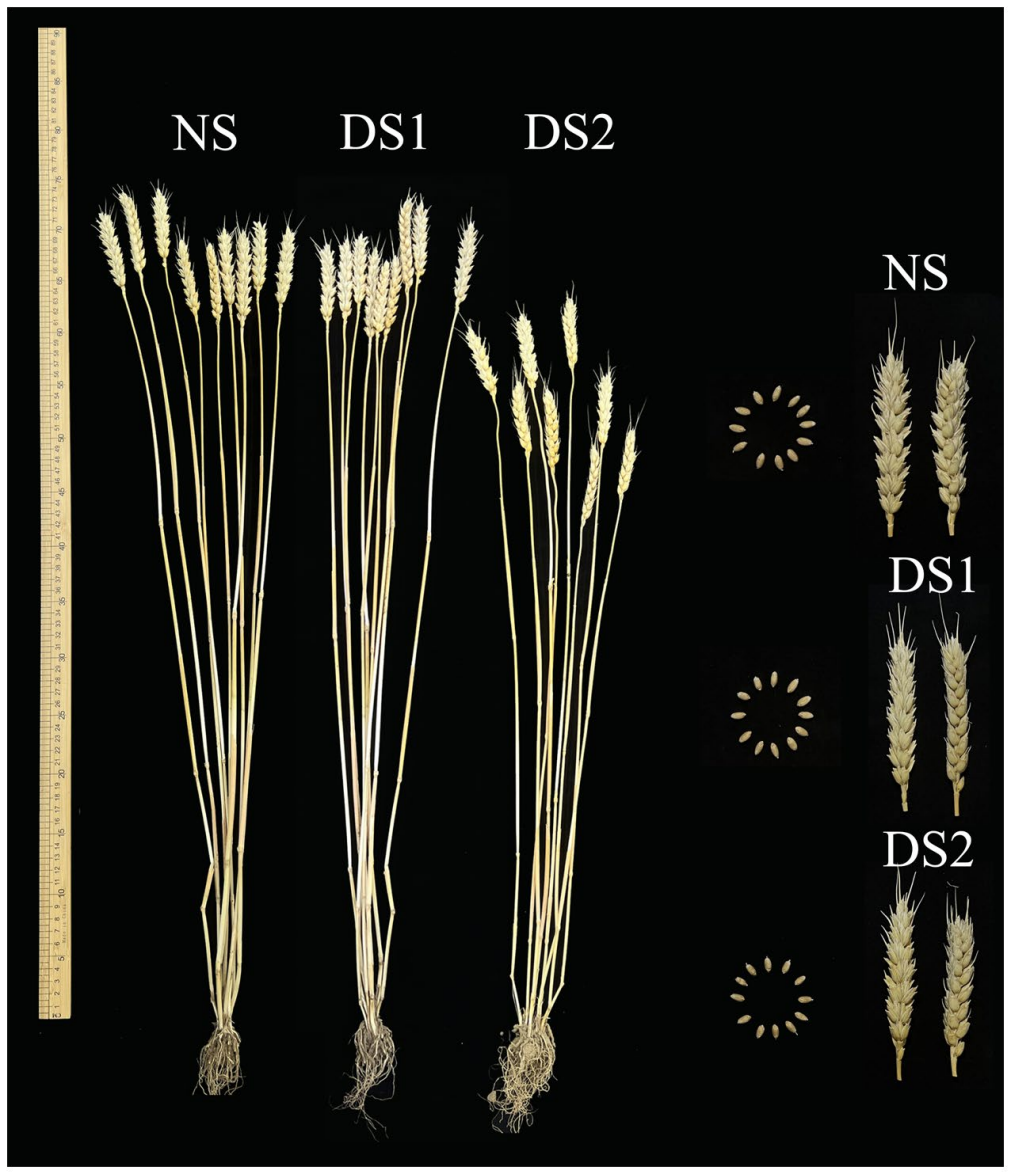
Agronomic and yield performance of the wheat cultivar Jinan 17 under normal sowing (NS), delayed sowing for 15 days (DS1), and delayed sowing for 30 days (DS2).

Compared with NS, the PH of the wheat cultivar Jinan 17 increased by 1.23% under DS1, but decreased by 12.79% under DS2 (Figure 6 and Table 4). The SL, SNPP, SNS, and KNS were all negatively affected by delayed sowing and decreased by 14.42, 6.52, 1.71, and 6.95%, respectively, under DS1. While under DS2, the SL, SNPP, SNS, and KNS were affected more severely, which decreased by 27.92, 10.86, 10.28, and 7.17%, respectively. Compared with the NS, the TKW increased by 3.66% under DS1, while decreased by 5.83% under DS2. In general, the wheat cultivar Jinan 17 showed enhanced TKW when sowing was postponed appropriately, but late sowing would deteriorate the agronomic and yield traits of the wheat cultivar Jinan 17.

**Table 4:** Agronomic and yield traits of the wheat cultivar Jinan 17 under normal sowing (NS), delayed sowing for 15 days (DS1), and delayed sowing for 30 days (DS2).

Treatment condition	PH (cm)	SL (cm)	SNPP	SNS	KNS	TKW(g)
NS	72.7 ± 2.0^a^	11.1 ± 0.6^a^	9.2 ± 0.6^a^	17.5 ± 0.7^a^	44.6 ± 3.2^a^	43.7 ± 0.1^a^
DS1	73.6 ± 3.1^a^	9.5 ± 0.6^b^	8.6 ± 0.5^ab^	17.2 ± 1.2^b^	37.5 ± 4.0^c^	45.3 ± 0.1^b^
DS2	63.4 ± 4.1^b^	8.0 ± 0.8^c^	8.2 ± 0.5^b^	15.8 ± 1.6^c^	41.4 ± 4.8^b^	40.8 ± 0.1^b^

Means with the same superscript in a column differ significantly (*p* ≤ 0.05). Displayed results are the means of two independent assays. Data represented as mean value ± SD based on the *chi*-squared test. PH, plant height; SL, spike length; SNPP, spike numbers per plant; SNS, spikelet numbers per spike; KNS, kernel numbers per spike; TKW, thousand-kernel weight.

### 3.8 Correlation analysis of starch physicochemical properties of wheat variety Jinan 17

**Figure 7:**
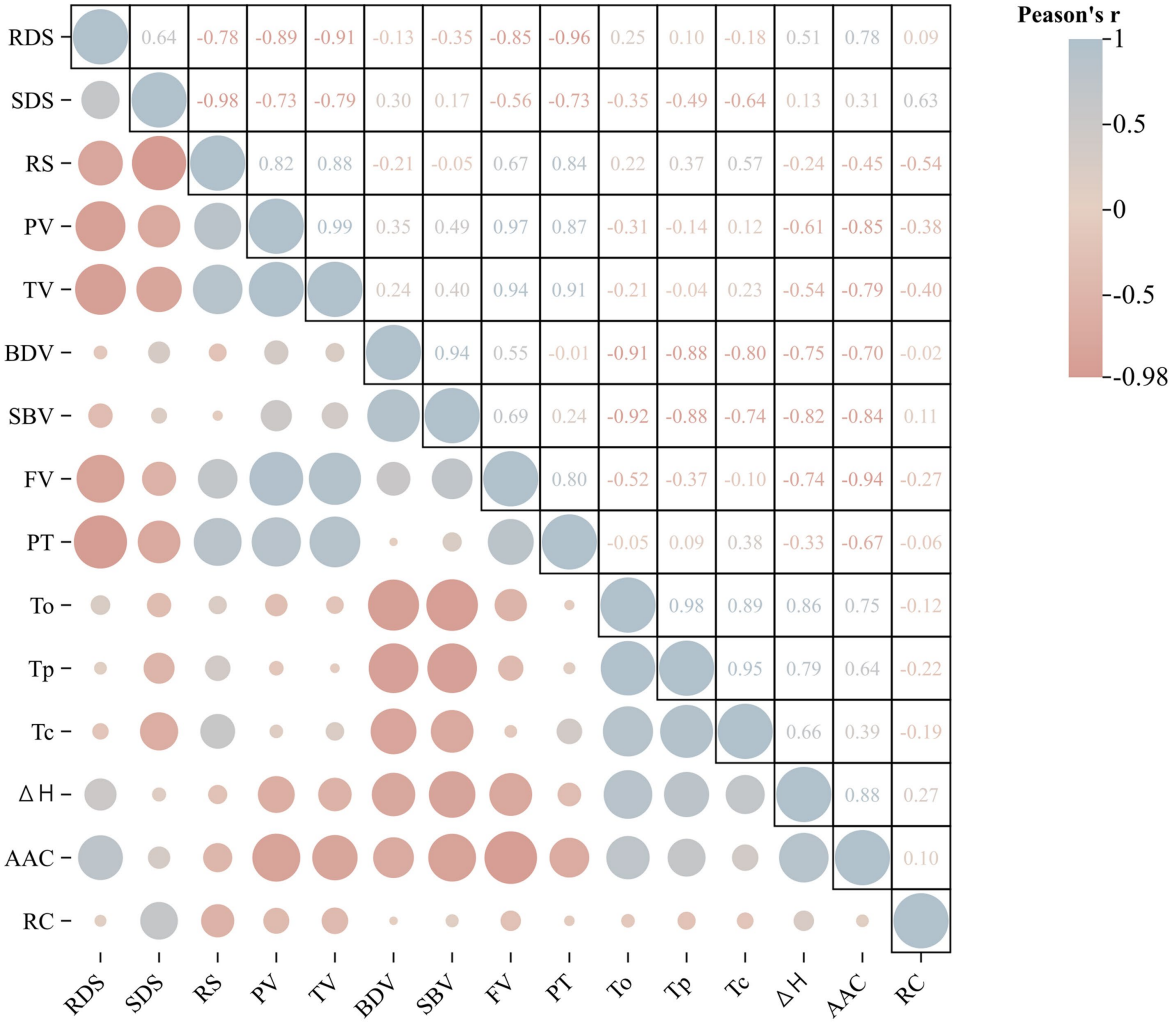
Correlation analysis of starch structure and physicochemical properties of the wheat cultivar Jinan 17 (JN17) starch samples under normal sowing (NS), delayed sowing for 15 days (DS1), and delayed sowing for 30 days (DS2) conditions. In the figure, blue represents positive correlation, red represents negative correlation, and the size of the circle represents the strength of the correlation. RDS, rapidly digestible starch; SDS, slowly digestible starch; RS, resistant starch; PV, peak viscosity; TV, hold-through viscosity; BDV, breakdown viscosity; SBV, setback viscosity; FV, final viscosity; PT, pasting temperature; *T*_o_, onset temperature; *T*_p_, peak temperature; *T*_c_, conclusion temperature; Δ*H*, gelatinization enthalpy; AAC, apparent amylose content; RC, relative crystallinity.

The correlation coefficient between starch structure and physicochemical properties is summarized (Figure 7 and Supplementary Table S1). The pairwise correlation analysis between AAC and pasting properties showed that AAC was significantly negatively correlated with PV, SBV, and TV. At the same time, it was significantly positively correlated with ∆H. In the pasting properties, there was a significant correlation between the most relevant indicators. The results showed that PV was significantly positively correlated with TV, FV, and PT. TV was significantly positively correlated with FV and PV. There was a significant negative correlation between BDV, SBV, and thermal properties, such as *T*_o_, *T*_p_, and ∆*H*. In the correlation analysis between starch structure and physicochemical properties, it was found that there was a significant positive or negative correlation between starch digestion characteristics and other physicochemical properties. RDS was significantly negatively correlated with PV, TV, FV, and PT. RS was positively correlated with PV, TV, and PT. There was a significant negative correlation between SDS and RS in digestive characteristics.

## 4 Discussion

Starch quality traits are important evaluation indexes of high-quality wheat. However, the formation of quality traits is a complex and finely regulated process, which is influenced by a set of factors, such as the genetic background mainly decided by cultivar’s differences and impact due to environmental factors. The latter, in particular, received more attention with the increasing climate changes globally, and the production of high-quality wheat faced severe challenges in fighting extreme climate. More adaptability and mechanism behind it are desiderated to be clarified. To adapt to these changes, the adjustment of sowing date, especially delayed sowing, is a common means. To analyze the effect caused by delayed sowing, in this study, a high-quality wheat cultivar for bread making Jinan 17 was selected to confirm the effects of delayed sowing conditions on the starch properties, which play a key role in the processing quality of the starch.

### 4.1 Expression patterns of the SS genes and starch physicochemical properties under different sowing dates

First, starch synthesis is a delicate and complex process controlled by a large number of genes through multiple pathways. To investigate the effect of delayed sowing on wheat starch, we determined the changes in starch properties at different levels and the expression levels of 10 key genes for SS. Under delayed sowing, AAC and RC were reduced. On the one hand, the change of RC may be due to the influence of granule structure or microcrystalline arrangement in the crystalline region of wheat starch, which is not conducive to the formation of the internal crystalline region. On the other hand, it may be caused by the change in amylopectin structure (30–33). Correspondingly, the expression levels of *SSIIIa*, *BEIIb*, and *GBSSI* changed significantly during endosperm development under delayed sowing conditions. Previous studies indicated that the suppression of *BEIIb* or the mutation of branched amylose can cause a longer length of the inner chain than that of normal amylose, whereas the frequency of branched amylopectin’s outer chain will be reduced (34). Thus, the change in RC in the present study may be due to the downregulated expression of *SSIIa*, leading to a decrease in the amylose content, a decrease in the distribution of branched amylose chain lengths, and an alteration in the granule morphology. This is also consistent with the previous reports (34). Meanwhile, we speculated that the decrease in AAC may be caused by the decrease in the expression level of *GBSSI*. A significant negative correlation was found between the pasting characteristics and AAC in the present study (Figure 7 and Supplementary Table S1), and the AAC has a significant effect on the pasting characteristics of starch (35). Therefore, delayed sowing may be the reason for the decrease in the AAC of starch, which promotes the swelling of starch granules, thus increasing the peak viscosity. This is also consistent with the results of the present study (36). The contribution of starch granules to an increase in starch viscosity was also documented in the previous study (37). Additionally, the enhancement of starch pasting properties may be due to the higher temperature encountered in the flowering and filling stages during the delayed sowing period than in the normal sowing period. The increase in pasting properties may be due to the increase in the long-chain content of amylopectin due to the high temperature encountered (Supplementary Figure S1 and Supplementary Table S2).

### 4.2 Morphological structure and *in vitro* digestion of starch under different sowing dates

**Figure 8:**
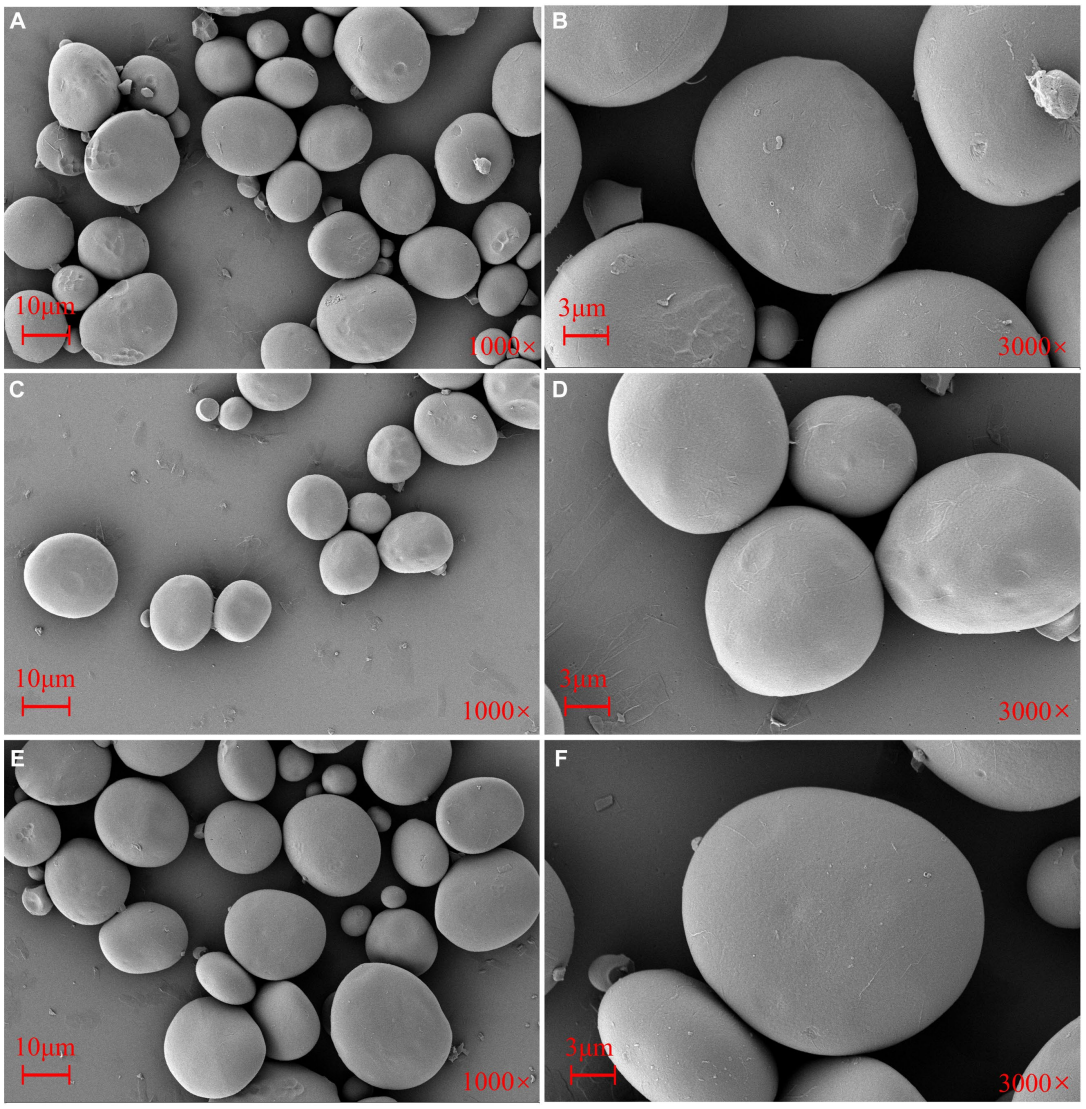
Scanning electron microscope (SEM) photographs of the starch granules from the wheat cultivar Jinan 17 under normal sowing **(A,D)**, delayed sowing for 15 days **(B,E)**, and delayed sowing for 30 days **(C,F)**. Scales in **A–C** = 10 μm, the magnification is 1,000×. Scales in **D–F** = 3 μm, and the magnification is 3,000×.

Another interesting phenomenon was that delayed sowing increased the content of RS. The surface of wheat starch under NS had fragments and some incomplete granules with uneven surface pits, whereas the surface of starch granules was smoother under delayed sowing (Figure 8). Surface morphology and the granule size of starch were also related to starch digestibility (39). In general, a larger specific surface area was found to be typical of granules with smaller sizes, resulting in enhanced contact between enzymes and starch to facilitate digestion (39). However, smaller granule size corresponds to higher sensitivity to enzymes regardless of plant origin, which could explain the increase in RS content after delayed sowing. The surface morphology of the particles was different, which led to structural differences. In addition, surface pores and internal channels existed in different sizes, strengths, and distributions. The functional nature of starch granules also implied that larger pores and channels allow water, chemicals, and enzymes to enter the interior of the granule more easily, thus improving starch pasting characteristics (39, 40).

### 4.3 Agronomic and yield changes under different sowing dates

Apart from the effects on the starch properties, delayed sowing may also bring unknown associated impacts on other traits, such as agronomic and yield traits, because it is a coordinated expression process between different traits in wheat. Generally, delayed sowing deteriorated the agronomic and yield traits of the wheat cultivar Jinan 17. The sowing date has a greater impact on the flowering period, maturity, and grouting duration, such as shortening the flowering and grain-filling periods. In particular, delayed sowing will lead to unfavorable weather at the unsuitable growing stage, such as dry-hot wind in the grain-filling, which can seriously reduce the TKW. As for the wheat cultivar Jinan 17, delayed sowing caused a significant reduction in SL, SNPP, SNS, and KNS. However, the PH increased under DS1 because delayed sowing most likely improved the synthesis and accumulation of lignin and cellulose in wheat and increased stem fullness based on the previous study (41). Delayed sowing increased wheat PH and lodging resistance. According to previous reports, the improvement of lodging resistance is mainly achieved by reducing the height of the stem center of gravity and increasing the tensile strength of basal internodes (42).

## 5 Conclusion

In this study, we investigated the effects on the starch properties and agronomic and yield traits under delayed sowing: (1) delayed sowing may indirectly affect the processing quality of the starch by affecting the starch crystallinity; (2) the changes in thermal properties of starch caused by delayed sowing can provide a reference for predicting and controlling starch properties during food processing; (3) appropriately delayed sowing can improve the pasting characteristics of the starch, but excessive delay in sowing time may reverse the effect; (4) delayed sowing date can regulate nutritional value and health benefits by affecting starch digestion characteristics; (5) behind a series of phenotypic changes in starch properties may be controlled by the adjustment of multiple SS genes in related metabolic pathways and regulatory networks; (6) agronomic and yield traits were also significantly affected that were associated with the changes in starch properties under delayed sowing.

Therefore, the effects of delayed sowing on high-quality wheat are multifaceted and complex, involving multiple dimensions from fine structure, and functional properties of the starch to the regulation of key gene expression. Dissecting the underlying mechanisms holds significant practical value for optimizing wheat planting management and maximizing the potential in terms of both quality and yield.

## Data availability statement

The original contributions presented in the study are included in the article/Supplementary material, further inquiries can be directed to the corresponding authors.

## Author contributions

XH: Data curation, Formal analysis, Methodology, Validation, Writing – original draft. XZ: Data curation, Formal analysis, Methodology, Supervision, Validation, Writing – original draft. XuL: Data curation, Formal analysis, Methodology, Writing – review & editing. WZ: Data curation, Formal analysis, Validation, Writing – review & editing. XW: Formal analysis, Validation, Writing – review & editing. ZJ: Data curation, Validation, Writing – review & editing. YY: Data curation, Validation, Writing – review & editing. QX: Data curation, Validation, Writing – review & editing. NL: Data curation, Validation, Writing – review & editing. XiL: Data curation, Validation, Writing – review & editing. YJ: Data curation, Validation, Writing – review & editing. GW: Conceptualization, Supervision, Validation, Writing – review & editing. JW: Conceptualization, Project administration, Resources, Supervision, Validation, Writing – review & editing. PM: Conceptualization, Project administration, Resources, Supervision, Validation, Writing – review & editing.
