# Supplementary material for: Structural and physicochemical effects on the starch quality of the high-quality wheat genotype caused by delayed sowing
Source: Front Nutr. 2024 Apr 16;11:1389745. doi: 10.3389/fnut.2024.1389745 (PMC11058212; doi:10.3389/fnut.2024.1389745)
Supplement: Supplementary file 1 [file Table_1.DOCX]

**TABLE S1** Parameters comparisons about starch structure and physicochemical properties of the wheat cultivar Jinan 17 (JN17) starch samples under normal sowing (NS), delayed sowing for 15 days (DS1) and delayed sowing for 30 days (DS2) conditions. RDS: Rapidly digestible starch; SDS: Slowly digestible starch; RS: Resistant starch; PV, Peak viscosity; TV: Hold-through viscosity; BDV: Breakdown viscosity; SBV: Setback viscosity; FV: Final viscosity; PT: Pasting temperature; To: Onset temperature; Tp: Peak temperature; Tc: Conclusion temperature; ΔH: Gelatinization enthalpy; AAC: Apparent amylose content; RC: Relative crystallinity.

|  | RDS | SDS | RS | PV | TV | BDV | SBV | FV | PT | To | Tp | Tc | ΔH | AAC | RC |
| --- | --- | --- | --- | --- | --- | --- | --- | --- | --- | --- | --- | --- | --- | --- | --- |
| RDS | 1 | 0.641 | -0.779 | -.890^*^ | -.906^*^ | 0.126 | -0.351 | -.845^*^ | -.956^**^ | 0.251 | 0.105 | 0.182 | 0.513 | 0.780 | 0.088 |
| SDS | 0.641 | 1 | -.981^**^ | 0.730 | 0.793 | 0.301 | 0.173 | -0.556 | -0.734 | -0.349 | 0.491 | 0.641 | 0.133 | 0.307 | 0.629 |
| RS | -0.779 | -.981^**^ | 1 | .824^*^ | .880^*^ | 0.214 | -0.051 | 0.670 | .844^*^ | 0.221 | 0.374 | 0.570 | 0.240 | -0.450 | 0.536 |
| PV | -.890^*^ | -0.730 | .824^*^ | 1 | .993^**^ | 0.347 | 0.494 | .968^**^ | .873^*^ | -0.309 | 0.144 | 0.123 | 0.613 | -.847^*^ | 0.384 |
| TV | -.906^*^ | -0.793 | .880^*^ | .993^**^ | 1 | 0.236 | 0.396 | .936^**^ | .906^*^ | -0.208 | 0.040 | 0.226 | 0.542 | -0.792 | 0.396 |
| BDV | -0.126 | 0.301 | -0.214 | 0.347 | 0.236 | 1 | .944^**^ | 0.547 | -0.011 | -.912^*^ | -.884^*^ | 0.801 | 0.752 | -0.696 | 0.015 |
| SBV | -0.351 | 0.173 | -0.051 | 0.494 | 0.396 | .944^**^ | 1 | 0.694 | 0.242 | -.924^**^ | -.882^*^ | 0.736 | -.818^*^ | -.844^*^ | 0.112 |
| FV | -.845^*^ | -0.556 | 0.670 | .968^**^ | .936^**^ | 0.547 | 0.694 | 1 | 0.803 | -0.517 | 0.370 | 0.105 | 0.739 | -.944^**^ | 0.267 |
| PT | -.956^**^ | -0.734 | .844^*^ | .873^*^ | .906^*^ | 0.011 | 0.242 | 0.803 | 1 | -0.051 | 0.090 | 0.375 | 0.334 | -0.675 | 0.058 |
| To | 0.251 | -0.349 | 0.221 | 0.309 | 0.208 | -.912^*^ | -.924^**^ | -0.517 | -0.051 | 1 | .984^**^ | .894^*^ | .861^*^ | 0.750 | 0.122 |
| Tp | 0.105 | -0.491 | 0.374 | 0.144 | 0.040 | -.884^*^ | -.882^*^ | -0.370 | 0.090 | .984^**^ | 1 | .952^**^ | 0.787 | 0.635 | 0.218 |
| Tc | -0.182 | -0.641 | 0.570 | 0.123 | 0.226 | 0.801 | -0.736 | -0.105 | 0.375 | .894^*^ | .952^**^ | 1 | 0.660 | 0.393 | 0.187 |
| ΔH | 0.513 | 0.133 | -0.240 | 0.613 | 0.542 | 0.752 | -.818^*^ | -0.739 | -0.334 | .861^*^ | 0.787 | 0.660 | 1 | .881^*^ | 0.267 |
| AAC | 0.780 | 0.307 | -0.450 | -.847^*^ | 0.792 | 0.696 | -.844^*^ | -.944^**^ | -0.675 | 0.750 | 0.635 | 0.393 | .881^*^ | 1 | 0.104 |
| RC | 0.088 | 0.629 | -0.536 | 0.384 | 0.396 | 0.015 | 0.112 | -0.267 | -0.058 | -0.122 | 0.218 | 0.187 | 0.267 | 0.104 | 1 |

**Note**: * And ** indicate signicant at p<0.05 and 0.01 levels, respectively.
