# Supplementary figures and images for: Structural and physicochemical effects on the starch quality of the high-quality wheat genotype caused by delayed sowing
Source: Front Nutr. 2024 Apr 16;11:1389745. doi: 10.3389/fnut.2024.1389745 (PMC11058212; doi:10.3389/fnut.2024.1389745)

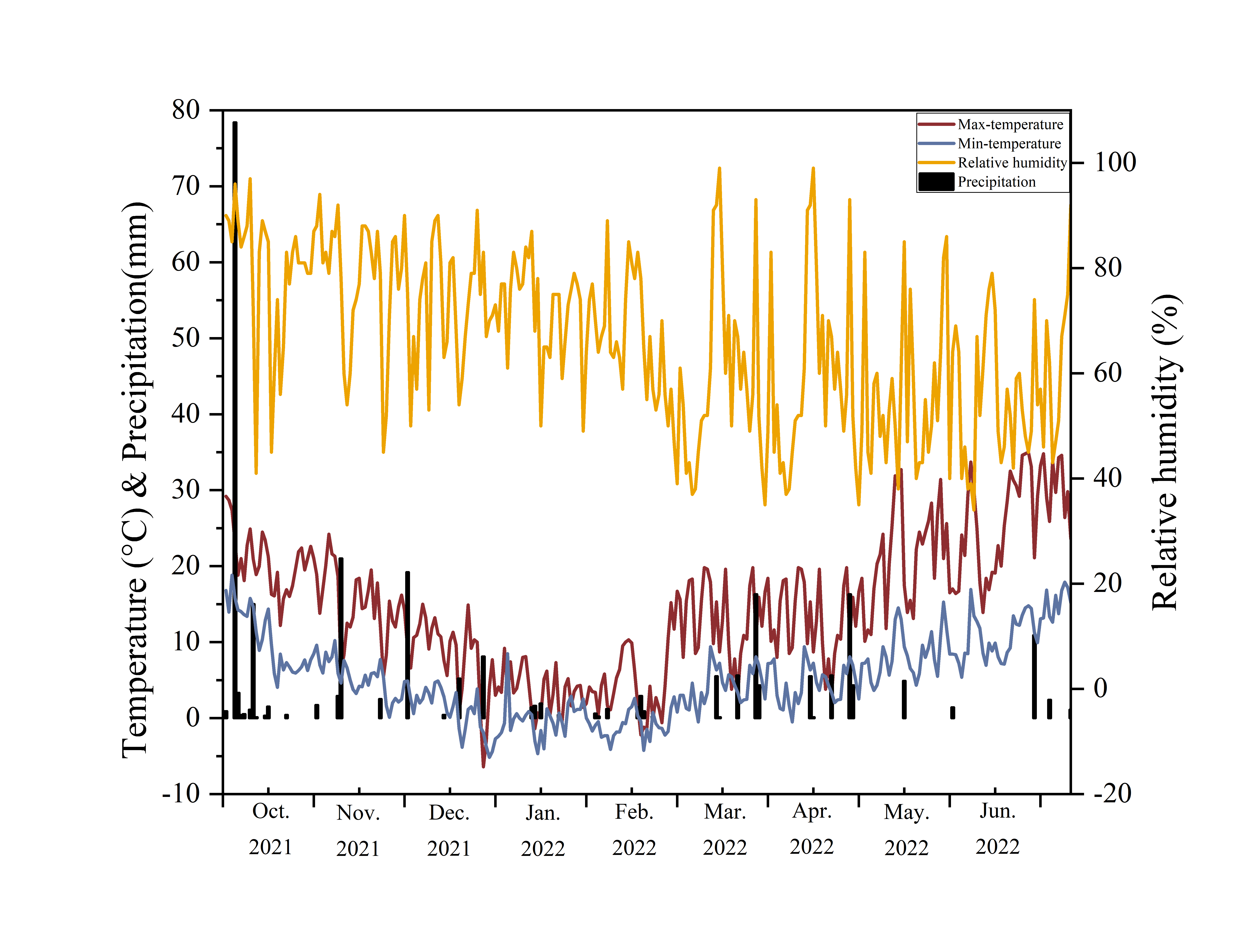

Supplement: Supplementary file 3 [file Image_1.TIF]
